# Circular RNA hsa_circ_0007507 May Serve as a Biomarker for the Diagnosis and Prognosis of Gastric Cancer

**DOI:** 10.3389/fonc.2021.699625

**Published:** 2021-09-14

**Authors:** Weiwei Zhang, Ming Zheng, Shan Kong, Xian Li, Shuting Meng, Xudong Wang, Feng Wang, Chenxue Tang, Shaoqing Ju

**Affiliations:** ^1^Department of Laboratory Medicine, Affiliated Hospital of Nantong University, Nantong, China; ^2^Research Center of Clinical Medicine, Affiliated Hospital of Nantong University, Nantong, China; ^3^Nantong University School of Medicine, Nantong, China; ^4^Department of Laboratory Medicine, Hai’an People’s Hospital, Hai’an, China

**Keywords:** gastric cancer, GC, hsa_circ_0007507, biomarker, diagnosis

## Abstract

**Purpose:**

The morbidity and mortality of gastric cancer (GC) remain high worldwide. In recent years, circular RNAs (circRNAs) have attracted widespread attention among cancer researchers due to the stable ring structure. The present work aims to find serum circRNA biomarkers that can be used in clinical applications and effective diagnosis.

**Methods:**

Hsa_circ_0007507 was extracted through circRNA sequencing. Exonuclease digestion assay, actinomycin D, agarose gel electrophoresis (AGE), and Sanger sequencing verified the potential of hsa_circ_0007507 as a biomarker. Besides, a real-time fluorescent quantitative polymerase chain reaction (RT-qPCR) was established to detect the level of expression of hsa_circ_0007507. Twenty cases of GC and the paired adjacent tissues were collected to verify its overexpression. Then, serum samples from 30 cases of colorectal cancer, 30 cases of thyroid cancer, and 30 cases of breast cancer were collected to verify their organ specificity. Additionally, serum samples from 80 healthy people, 62 gastritis patients, 31 intestinal metaplasia patients, and 100 GC patients were collected, and the diagnostic efficacy was evaluated through analysis of the receiver operating characteristic (ROC) curve. Furthermore, 16 post-operative GC samples, samples of 65 relapsed patients and 36 non-relapsed patients were collected to evaluate the prognosis of GC.

**Results:**

The level of expression of hsa_circ_0007507 in GC tissues was up-regulated (*p* = 0.0121), which was consistent with the results of circRNA sequencing. Exonuclease digestion assay and actinomycin D confirmed that hsa_circ_0007507 had a stable structure and a longer half-life. In the analysis of organ specificity experiments, serum hsa_circ_0007507 did not have specificity for patients with colorectal cancer (*p* = 0.5319), thyroid cancer (*p* = 0.5422), or breast cancer (*p* = 0.5178). Analysis of diagnostic efficacy indicated that the expression of hsa_circ_0007507 was significantly higher than that of normal people (*p <*0.0001); the area under the ROC (AUC) was 0.832 (95% CI: 0.771-0.892); the diagnostic power of hsa_circ_0007507 was higher than that of CEA (AUC = 0.765, 95% CI: 0.697-0.833) and CA199 (AUC = 0.587, 95% CI: 0.504-0.67). Through diagnosis using a combination of the three, GC patients could be distinguished from normal people (AUC = 0.849), and higher diagnostic efficiency could be achieved. The expression of serum hsa_circ_0007507 in GC patients significantly decreased after surgery (*p* = 0.001). Besides, the expression of serum hsa_circ_0007507 in patients with post-operative recurrence was significantly up-regulated again (*p* = 0.0139).

**Conclusions:**

Serum hsa_circ_0007507 is differentially expressed in GC patients, post-operative GC patients, gastritis patients, intestinal metaplasia patients and relapsed patients, suggesting that serum hsa_circ_0007507 can be used as a new diagnostic and dynamic monitoring biomarker for GC.

## Introduction

GC remains among those cancers with the highest incidence in the world, ranking third in the list of global causes of cancer-related death (1). Generally, patients with early GC have no obvious symptoms and are at an advanced stage when diagnosed (2). Since GC has high rate of recurrence and is prone to distant metastasis, it still has the worst consequence among all solid organ tumors. Therefore, it is necessary to find new diagnostic methods, evaluate prognostic indicators, and determine its characteristic biological targets.

Non-coding RNA (ncRNA) is the general term for RNA that is not involved in protein coding. According to the Human Genome Sequencing Project, scientists found that the genes encoding proteins account for only a small number thereof, and about 80% of the transcripts are ncRNAs (3). These have long been considered “useless” transcription products. Besides, they are currently confirmed to play an essential role in maintaining normal physiological functions of the body (4–6) and the normal metabolism of cells (7, 8); moreover, they can regulate the proliferation, invasion, and migration of GC cells through different mechanisms (9–11), providing a potential target for the treatment of GC. In recent years, the non-coding RNAs that are most relevant to the diagnosis (12) of GC are mainly circular RNA, long-chain non-coding RNA (1ncRNA), and small RNA (mirco-RNA).

CircRNAs are a type of single-stranded RNA with developmental/tissue-specific expression patterns in eukaryotic cells. CircRNAs are produced by “reverse splicing” of the pre-mRNA transcript, forming a covalently closed loop-structure without 5’ and 3’ polarity or a 3’ polyA tail structure (13). The unique circular structure of the circRNA equips it with inherent resistance to exonuclease, making it more stable than the linear mother gene. It has been verified that circRNAs are abnormally expressed in tumor tissues and cells and play an indispensable role in the occurrence and development of various tumors, such as hepatocellular carcinoma (HCC), lung cancer, GC, breast cancer, bladder cancer, and glioblastoma (14–19). Therefore, circRNAs have great potential as valuable diagnostic biomarkers or therapeutic targets. CircRNAs usually exhibit different functions according to their location in the cell. Most circRNAs are derived from gene exons, called exon circRNAs (ecircRNAs), located in the cytoplasm. circRNAs can regulate gene expression at the transcriptional level by combining miRNA as a molecular sponge and bind to RNA-binding proteins and interfere with the normal function of genes after transcription. The other two circRNAs, intron circRNA (ciRNA) and exon-intron circular RNA (EIciRNA), are mostly located in the nucleus, playing a regulatory role in the transcription of their mother gene or serving as a template for protein translation (20). These findings provide new directions for circRNAs as targeted molecules for disease diagnosis and prognosis.

To ascertain those differentially expressed circRNAs, we used high-throughput sequencing to detect the expression of circRNAs in three pairs of GC tissues and identified 2007 significantly different circRNAs through circRNA sequencing. Subsequently, hsa_circ_0007507 was selected as the research object to further investigate serum samples from 100 GC patients and 80 normal people. Besides, the clinical utility of hsa_circ_0007507 in GC diagnosis was evaluated through receiver operating characteristic (ROC) curve analysis to provide a novel biomarker for GC research.

## Materials and Methods

### Specimen Collection

Participants in this study were either patients or healthy people who came for physical examination in the Affiliated Hospital of Nantong University (Nantong, China). Twenty pairs of GC and corresponding adjacent tissues were collected. In addition, peripheral blood serum samples of the following different populations were collected: newly diagnosed GC patients (100), superficial gastritis patients (30), atrophic gastritis patients (32), intestinal metaplasia patients (31), colorectal cancer patients (30), thyroid cancer patients (30), breast cancer patients (30) and age-matched healthy controls (80). Besides the above GC patients (100) who were pathologically or clinically diagnosed and had not been subjected neither to surgery related to the present study, nor chemotherapy or radiotherapy were collected, as well as peripheral blood serum samples of post-operative recurrence patients (65) and corresponding post-operative GC patients (16). All samples were stored at -80°C in a refrigerator until subsequent processing. The study was conducted under the approval of the Ethics Committee of Affiliated Hospital of Nantong University (ethical review report number: 2018-L055).

### Cell Culture

Human GC cell lines (MKN-45, SGC-7901, HGC-27, BGC-823, MKN-1, and AGS) and human gastric epithelial cells (GES-1) were purchased from the Stem Cell Bank of the Chinese Academy of Sciences (Shanghai, China), among which GES-1 was taken as the normal control. The cells were cultured in RPMI-1640 medium (Corning, Manassas, VA), which was subsequently supplemented with 10% Fetal bovine serum (FBS, Gibco, Grand Island, NY) and 1% penicillin and streptomycin, in a humidified incubator at 37°C and with 5% CO2.

### Total RNA and gDNA Extraction

The total RNA in cells and serum was extracted by TRIzol reagent (Invitrogen, Karlsruhe, Germany) and TRIzol LS reagent (Invitrogen, Canada), respectively. Then, their concentration and purity were detected by NanoDropTM One (Thermo Fisher Scientific, USA). Besides, genomic DNA (gDNA) was extracted from cells using the Revert Aid First Strand cDNA Synthesis Kit (Thermo Fisher Scientific, MA, USA).

### Reverse Transcription and Real-Time Fluorescent Quantitative PCR

The total RNA was reverse-transcribed into cDNA by a reverse transcription kit (Thermo Fisher Scientific, MA, USA) after extraction, under the following the protocols: 25°C for 5 minutes, 42°C for 60 minutes, and 70°C for 5 minutes. RT-qPCR was conducted using LightCycler ^®^ 480 (Roche, Switzerland) with the 2^−ΔΔCT^ relative quantitative calculation method. The housekeeping gene GAPDH and 18S, whose primers were synthesized by Sangon Biotech Corporation (Shanghai, China), were taken as the internal control for cells and serum samples, respectively. The specific divergent primers used in this study were synthesized by RiboBio Corporation (Suzhou, China). The sequences of the involved gene included: hsa_circ_0007507: AGTAGAAGAGGTGGTGGTATCA (forward) and GGCCTCCGATCACTCTCTCT (reverse); RASA1: CAGGGAAGTCTGGCAGTTATCT (forward) and TCTCCACACATAGCAATAATCCT (reverse); GAPDH: AGAAGGCTGGGGCTCATTTG (forward) and GCAGGAGGCATTGCTGATGAT (reverse); 18S: CGGCTACCACATCCAAGGAA (forward) and GCTGGAATTACCGCGGCT (reverse). Besides, PCR products were stored at -20°C in a refrigerator until subsequent Sanger sequencing and AGE.

### RNase R and Actinomycin D Assays

Total RNA (10 μg) extracted from BGC-823 and MKN-1 cells was treated with Ribonuclease R (RNase R, 3-4U/μg) from Geneseed Biotech Co., Ltd (Guangzhou, China). The reaction system was then incubated at 37°C for 1 minute, followed by incubation at 70°C for 10 minutes, to inactivate the enzyme and terminate the reaction for subsequent reverse transcription. Next, 1 mg/ml actinomycin D was diluted into 2.5 μg/ml actinomycin D with a complete medium, and the ordinary medium in the six-well plate was replaced with the treated complete medium. Cellular RNA was extracted at 0h, 2h, 4h, 8h, and 12h, respectively.

### Nucleoplasm Separation Assay

The nuclear and cytoplasmic RNA of SGC-7901and BGC-823 cells were separated and isolated using a PARISTM Kit (Thermo Fisher Scientific) according to procedures in the manual. After trypsinization, the cells were counted to obtain a cell suspension of up to 10^7^, whose culture medium was discarded after centrifugation at low speed. The cell pellet was washed once with PBS, and subsequent operations on ice were performed. Besides, the cells with 100 to 500μl pre-chilled cell fractionation buffer were resuspended, followed by incubation on ice for 5-10 minutes. Then, they were centrifuged at 500*g* for 1-5min at 4°C, after which the cytoplasm was in the supernatant while the nucleus was in the pellet. The supernatant containing cytoplasm was transferred to a new tube for RNA extraction. The pellet containing nucleus was subsequently mixed with Cell Disruption Buffer, using the same volume as that of Cell Fractionation Buffer. The resulting mixture was vortexed until the lysate was uniform, thus obtaining the nucleus. The nuclear and cytoplasmic RNA were extracted according to the following instructions. An equal amount of 2× Lysis/Binding Solution was added and mixed at room temperature. Immediately gently pipet 3-4 times or invert the tube to mix well. Then, an equal amount of Ethanol absolute (ACS Grade) was added and fully mixed. The 700µl mixture was transferred into the adsorption column at a time and centrifuged at 12000 rpm for 1min. It was washed once with 700µl *Wash Solution 1* and twice with 500µl *Wash Solution 2/3*, before getting centrifuged for 30s to remove any residue. The filter element was placed in the attached clean collection tube, while the RNA was eluted twice (40μl and 20μl respectively) with the Elution Solution preheated at 95-100°C and then centrifuged at 12000 rpm for 30s. Finally, the separated nuclear and cytoplasmic RNA solution were obtained, for storage at -80°C.

### Luciferase Reporter Assay

Selected for Luciferase reporter assay, MKN-1 and BGC-823 cells were inoculated in 24-well plates when grown into the third generation. Cell transfection was conducted when the fusion reached the range of 30%~40%. The hsa_circ_0007507 WT plasmid and several mimics of microRNA, with the predicted binding relationship with hsa_circ_0007507, were designed by Ribo. After 48 hours, Luciferase reporter assay was performed according to the protocols of the kit as follows. Firstly, 100μl of LAR II was added to the 96-well white plate, and then 20μl of the PLB lysed cell lysate was added to measure firefly fluorescence. Finally, 100μl Stop & Glo Reagent was added to detect Renilla fluorescence. Through a comparison of the relative fluorescence of the firefly to the Renilla fluorescence with the control well, the binding relationship between the molecules was verified.

### Statistical Analysis

As for the analysis of sequencing results, the reads were mapped to the latest UCSC transcript set by Bowtie 2 version 2.1.0, and the gene level of expression was estimated utilising RSEM v1.2.15, which was then normalized by the trimmed mean *M*-value (TMM). Genes which were differentially expressed were then identified using the edgeR program. Those showing altered expression with more than two-fold changes were considered to be differentially expressed. SPSS 26.0 (IBM SPSS Statistics, Chicago, USA) and GraphPad Prism 9.0 (GraphPad Software, La Jolla, CA) were used for statistical analysis. The analysis of relative hsa_circ_0007507 expression was conducted using GraphPad Prism 9.0 using Student’s *t*-test. The unpaired *t*-test was used to analyze the difference of expression between patients with different diseases and the healthy controls while the paired *t*-test was used to analyze the difference of expression among the same patients before and after surgery. Moreover, one-way ANOVA was used when more than two groups of data were being compared. The ROC curve was plotted using SPSS, also playing a role in conducting chi-squared testing and Spearman correlation testing for analyzing the correlation with clinical features. A *P*-value of less than 0.05 is considered statistically significant. OS rate was evaluated by employing the Kaplan‐Meier method. P value < 0.05 was considered to be statistically significant.

## Results

### Method Verification of hsa_circ_0007507

**Figure 1:**
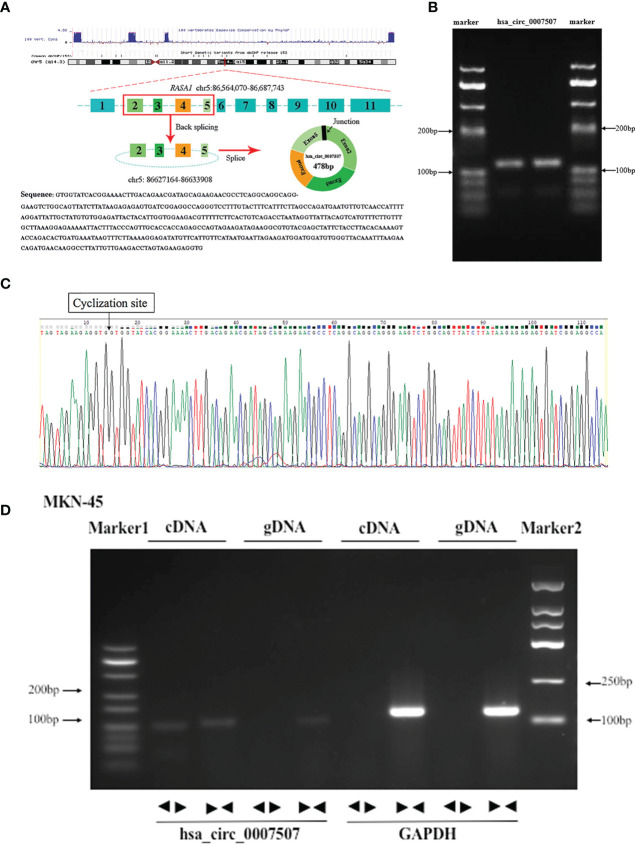
Methodological evaluation of hsa_circ_0007507 in gastric cancer (GC) cells. **(A)** The origin of hsa_circ_0007507 searched through the University of California Santa Cruz (UCSC) genome browser. **(B)** Verification of the size of the primer amplification product (112 bp) by agarose gel electrophoresis. **(C)** Detection of the cyclization site by Sanger sequencing. **(D)** Verification of the ring structure of hsa_circ_0007507.

High-throughput sequencing of three GC tissues and their matched noncancerous tissues was carried out to identify circRNAs aberrantly expressed in GC tissues (**Supplementary Files S1**, **S2**), from which hsa_circ_0007507 was selected. According to the Human circRNA Database (circbank, http://www.circbank.cn/index.html), hsa_circ_0007507 was located on chr5_86564070_86687743, and the length of its mature transcript was 478 bp. As analyzed by circPrimer software, hsa_circ_0007507 was composed of exons 2, 3, 4, and 5 (**Figure 1A**). Considering that circRNAs usually form a closed loop through reverse splicing, a divergent primer that can reverse-amplify the circular molecule was designed based on the hsa_circ_0007507 circularization site. The level of expression of hsa_circ_0007507 was then detected by RT-qPCR, the product of which was detected by 2% AGE. The electrophoresis band was 112 bp, which was consistent with the size of the primer amplified product (**Figure 1B**). The reverse splicing site of hsa_circ_0007507 was further determined using Sanger sequencing (**Figure 1C**). Then, RT-qPCR was performed with genomic DNA (gDNA) and cDNA as templates, and glyceraldehyde 3-phosphate dehydrogenase (GAPDH) as a negative control. Agarose electrophoresis revealed that hsa_circ_0007507 can be amplified from PCR products with cDNA as a template while negative results were observed in the control group with gDNA as a template (**Figure 1D**).

### The Characteristics of hsa_circ_0007507

**Figure 2:**
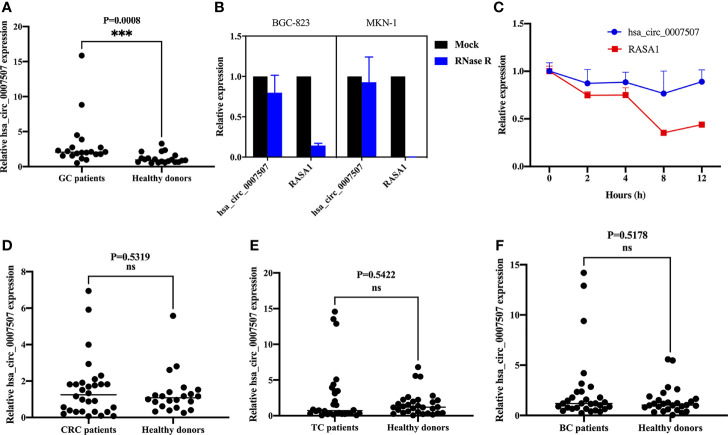
The characteristics of hsa_circ_0007507. **(A)** Differential expression of hsa_circ_0007507 in the serum of GC patients (n=20), and healthy donors (n=20). **(B)** Stability of hsa_circ_0007507 confirmed by Rnase R digestion assay. **(C)** Longer half-life of hsa_circ_0007507 verified by actinomycin D assay. **(D)** Differential expression of hsa_circ_0007507 in the serum of colorectal cancer patients (n=30), and healthy donors (n=22). **(E)** Differential expression of hsa_circ_0007507 in the serum of thyroid cancer patients (n=30), and healthy donors. (n=30). **(F)** Differential expression of hsa_circ_0007507 in the serum of breast cancer patients (n=30), and healthy donors. (n=27). Indicated statistical significance (***p < 0.001), ns indicated *p* > 0.05.

First, the expression of hsa_circ_0007507 was detected in 20 pairs of GC tissue and corresponding adjacent tissue samples by real-time quantitative PCR (RT-qPCR), and it was revealed to be significantly up-regulated in GC tissues (**Supplementary Files S3**). In order to explore its ability as a serum biomarker, the serum samples of 20 GC patients and 20 healthy donors were compared (**Figure 2A**). Many nucleases in cells can easily degrade RNA molecules while circRNAs have better stability and are not easily degraded. Therefore, BGC-823 and MKN-1 cells were treated with RNase R to verify the stability of hsa_circ_0007507. It was demonstrated that hsa_circ_0007507 was not easily degraded while its transcription gene RASA1 was severely degraded (**Figure 2B**). Besides, BGC-823 cells were cultured in a medium containing actinomycin D for 12 h, which can inhibit RNA production by inhibiting RNA polymerase. RT-qPCR showed that the half-life of hsa_circ_0007507 was significantly longer than the half-life of its transcription gene RASA1, further verifying the stability of hsa_circ_0007507 (**Figure 2C**). Furthermore, a large sample verification was conducted to verify the organ specificity of hsa_circ_0007507. Specifically, 30 serum specimens from patients with colorectal cancer were collected, and 22 normal human serum specimens were used as controls. The results of RT-qPCR indicated that the expression of hsa_circ_0007507 in colorectal cancer serum was not significantly different from that in normal human serum (*p* = 0.5319) (**Figure 2D**). Moreover, 30 serum samples from patients with thyroid cancer were collected, and 30 normal human serum samples were used as controls. The results of RT-qPCR proved that the expression of hsa_circ_0007507 in thyroid cancer serum was not significantly different from that in normal human serum (*p* = 0.5422) (**Figure 2E**). Moreover, 30 breast cancer serum samples were collected, and 27 normal human serum samples were used as controls. The results of RT-qPCR confirmed that the expression of hsa_circ_0007507 in breast cancer serum was not significantly different from that in normal human serum (*p* = 0.5178) (**Figure 2F**).

Considering the clinicopathological parameters of these patients, it can be concluded that the expression of hsa_circ_0007507 shows specificity in GC while the expression of colorectal cancer, thyroid cancer, and breast cancer is not statistically significant. Therefore, hsa_circ_0007507 has organ specificity in GC.

### The Diagnostic Value of hsa_circ_0007507

**Figure 3:**
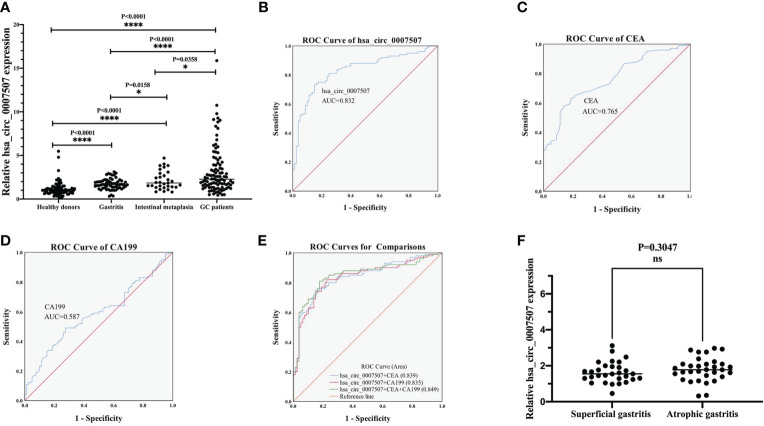
Evaluation of the diagnostic value of hsa_circ_0007507. **(A)** Differential expression of hsa_circ_0007507 in the serum of healthy donors (n=80), gastritis patients (n=62), intestinal metaplasia patients (n=31), and GC patients (n=100). **(B)** ROC curve analysis of serum hsa_circ_0007507 for discriminating GC patients and healthy donors (AUC=0.832). **(C)** The diagnostic efficacy of CEA (AUC=0.765). **(D)** The diagnostic efficacy of CA199 (AUC=0.587). **(E)** Combined diagnostic of serum hsa_circ_0007507, CEA and CA199 exerted the best diagnostic efficacy in distinguishing GC patients and healthy donors (AUC=0.849). **(F)** Differential expression of hsa_circ_0007507 in the serum of superficial gastritis patients (n=30) and atrophic gastritis patients (n=32). ROC, receiver operating characteristic curve; AUC, the area under the ROC. Indicated statistical significance (*p < 0.05, ****p < 0.0001).

A large sample verification was conducted to assess the diagnostic significance of hsa_circ_0007507 in gastritis, intestinal metaplasia, and GC serum. Serum samples from 100 GC patients and 80 healthy donors were collected for detecting the difference in their level of expressions. Since the early diagnosis of many patients with GC is difficult, and a considerable number of cases were misdiagnosed as from gastritis and intestinal metaplasia, it is particularly important for us to distinguish GC from gastritis. Then we also collected the serum samples from 30 cases of superficial gastritis, 32 cases of atrophic gastritis and 31 cases of patients with early gastric cancer lesions (intestinal metaplasia). We performed statistical analysis on the expression of hsa_circ_0007507 in the serum samples of normal people, gastritis, gastric cancer and intestinal metaplasia. Statistically, the expression of normal subjects was significantly different from that of the other three groups (gastritis, intestinal metaplasia and gastric cancer) (*p <*0.0001), and there were statistically significant differences between gastritis and intestinal metaplasia (*p* =0.0158) and gastric cancer (*p <*0.0001), as well as between intestinal metaplasia and gastric cancer (*p* =0.0358). The results showed that, as gastritis developed towards gastric cancer, the expression of hsa_circ_0007507 showed an increasing trend (**Figure 3A**).

To understand whether hsa_circ_0007507 can be used as a potential GC diagnostic marker, ROC curves of hsa_circ_0007507, CEA, and CA199 were analyzed, and the area under the curve (AUC) was calculated based on the data obtained. The ROC curve demonstrated that the AUC value of hsa_circ_0007507 was 0.832 (95% CI: 0.771-0.892, *p* < 0.001) (**Figure 3B**), higher than CEA (0.765, 95% CI: 0.697-0.833, *p* < 0.001) (**Figure 3C**), and CA199 (0.587, 95% CI: 0.504-0.67, *p* = 0.044) (**Figure 3D**). Particularly, the combined use of hsa_circ_0007507 and CEA produced an AUC of 0.839; the combined use of hsa_circ_0007507 and CA199 resulted in an AUC of 0.835; the combined use of the three produced the largest AUC of 0.849 (**Figure 3E**). Besides, hsa_circ_0007507 was higher than CEA and CA199 in terms of sensitivity (73%), specificity (85%), overall accuracy (78%), positive predictive value (86%), and negative predictive value (72%) (**Table 1**). This analysis suggested that the combined use can make up for the limitations of a single marker, and hsa_circ_0007507 as a biomarker has potential in the diagnosis of GC. Meanwhile, 30 cases of superficial gastritis and 32 cases of atrophic gastritis were statistically analyzed by t-test, and the results showed that there was no significant difference between the two groups(*p* =0.3047). However, the expression of hsa_circ_0007507 in atrophic gastritis samples was higher than that of superficial gastritis (**Figure 3F**).

**Table 1:** Evaluation of the diagnostic values of combination of hsa_circ_0007507, CEA, CA199 between GC patients and healthy donors.

	SEN,%	SPE,%	ACCU,%	PPV,%	NPV,%
hsa_circ_0007507	0.73 (73/100)	0.85 (68/80)	0.78 (141/180)	0.86 (73/85)	0.72 (68/95)
CEA	0.71 (71/100)	0.61 (49/80)	0.67 (120/180)	0.70 (71/102)	0.63 (49/78)
CA199	0.58 (58/100)	0.55 (44/80)	0.57 (102/180)	0.62 (58/94)	0.51 (44/86)
hsa_circ_0007507+CEA	0.75 (75/100)	0.85 (68/80)	0.79 (143/180)	0.86 (75/87)	0.73 (68/93)
hsa_circ_0007507+CA199	0.82 (82/100)	0.78 (62/80)	0.80 (144/180)	0.82 (82/100)	0.78 (62/80)
hsa_circ_0007507+CEA+CA199	0.81 (81/100)	0.83 (66/80)	0.82 (147/180)	0.85 (81/95)	0.78 (66/85)

SEN, sensitivity; SPE, specificity; ACCU, overall accuracy; PPV, positive predictive value; NPV, negative predictive value.

Then the 100 GC patients were divided into two groups [hsa_circ_0007507(high) and hsa_circ_0007507(low)] after the Youden index had been determined, which was calculated using SPSS software by importing the relative level of expressions of serum hsa_circ_0007507 in 100 GC patients and 80 healthy donors to evaluate the correlation between hsa_circ_0007507 expression and clinicopathological features in GC patients. As shown in **Table 2**, the level of expression of hsa_circ_0007507 was significantly related to tumor depth (*p* = 0.032*), lymph node metastasis (*p* = 0.028*), and TNM stage (*p* = 0.003**) using Pearson’s χ^2^ test, while there was no evidence that hsa_circ_0007507 expression was correlated with gender (*p* = 0.828), age (*p* = 0.517), tumor size (*p* = 0.1), differentiation grade (*p* = 0.464), or nerve/vascular invasion (*p* = 0.122).

**Table 2:** The association between hsa_circ_0007507 expression and the clinicopathological parameters in GC patients.

Parameter	No. of patients	hsa_circ_0007507(high)	hsa_circ_0007507(low)	P‐value
Sex				
Male	61	45	16	0.828
Female	39	28	11	
Age (years)				
<60	23	18	5	0.517
≥60	77	55	22	
Tumor size				
<5	57	38	19	0.1
≥5	43	35	8	
Differentiation grade				
Well-moderate	57	40	17	0.464
Poor-undifferentiation	43	33	10	
T stage				
T1–T2	53	31	18	0.032*
T3–T4	47	42	9	
Lymph node status				
Positive	38	23	15	0.028*
Negative	62	50	12	
TNM stage				
I–II	40	22	17	0.003**
III–IV	60	51	10	
Nerve/vascular invasion				
Positive	46	37	9	0.122
Negative	54	36	18	

Statistical analyses were carried out using Pearson χ2 test.

Indicated statistical significance (*p < 0.05, **p < 0.01).

### Prognostic Analysis of hsa_circ_0007507

**Figure 4:**
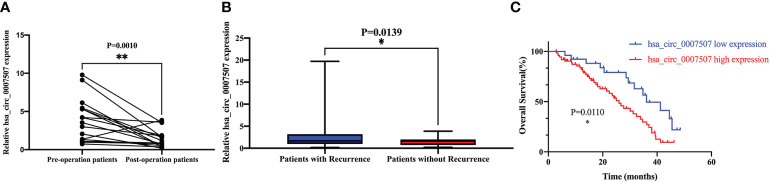
Prognostic analysis of hsa_circ_0007507. **(A)** Expression level of hsa_circ_0007507 in GC patients decreased after operation (n=16, p=0.0010). **(B)** Differential expression of hsa_circ_0007507 in the serum of recurrent GC patients (n=65), and non-recurrent patients (n=36). **(C)** The Kaplan–Meier survival curve verifies the prognostic value of hsa_circ_0007507. Indicated statistical significance (*p < 0.05, **p < 0.01).

Post-operative serum samples of 16 patients were collected one week after surgery to evaluate hsa_circ_0007507 expression. The level of expression of hsa_circ_0007507 decreased significantly after surgery (**Figure 4A**). The results verified that hsa_circ_0007507 can be used for cancer surveillance. Besides, 65 relapsed patients and 36 non-relapsed patients serum samples were collected. The results showed that the serum hsa_circ_0007507 expression in relapsed patients was higher than that in non-relapsed patients, with statistical significance (*p* =0.0139) (**Figure 4B**). The survival rate of the hsa_circ_0007507 (low) group was higher than that of the hsa_circ_0007507 (high) group (**Figure 4C**).

### Exploration of the Downstream Regulatory Network of hsa_circ_0007507 in GC Cells

**Figure 5:**
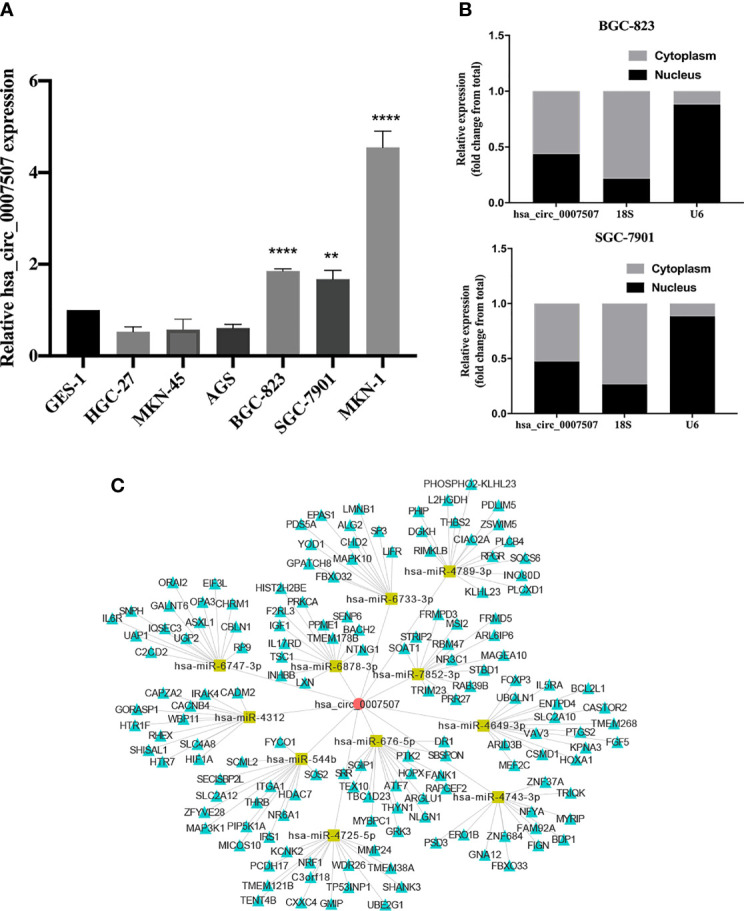
Exploration of the downstream regulatory network of hsa_circ_0007507 in gastric cancer (GC) cells. **(A)** Detection of hsa_circ_0007507 expression in six GC cell lines. **(B)** Detection of hsa_circ_0007507 location in BGC-823 and SGC-7901 cell lines by nucleoplasm separation assays. **(C)** Prediction of circular RNA (circRNA)-microRNA (miRNA)–messenger RNA (mRNA) network map of hsa_circ_0007507. The red round shape represents hsa_circ_0007507, and the yellow rectangle represents eleven miRNAs that could interact with hsa_circ_0007507, while the green triangle represents the target mRNA of the corresponding miRNA. Indicated statistical significance (**p < 0.01, ****p < 0.0001).

To investigate the functional mechanism of hsa_circ_0007507 in GC cell lines, normal gastric mucosal epithelial GES-1 cells were used as controls to detect the level of expression of hsa_circ_0007507 in six GC cell lines (HGC-27, MKN-45, SGC-7901, MKN-1, AGS, and BGC-823). Besides, the expression of hsa_circ_0007507 was significantly up-regulated in BGC-823, and MKN-1, and SGC-7901, and down-regulated in HGC-27, MKN-45, and AGS (**Figure 5A**). Furthermore, RNA was extracted from BGC-823 and SGC-7901 through nuclear and cytoplasmic separation. It was revealed that hsa_circ_0007507 accounted for a higher proportion of the cytoplasm. The FISH assay also showed that hsa_circ_0007507 was mainly distributed in the cytoplasm of BGC-823 cell line (**Supplementary Files S4**). indicating that it may participate in the process of GC mainly through post-transcriptional regulation (**Figure 5B**). Next, the potential circRNA-miRNA-mRNA regulatory axis in GC was predicted using bioinformatics database analysis (miRanda, PITA, TargetScan). As illustrated in **Figure 5C**, 11 miRNAs (hsa-miR-676-5p, hsa-miR-544b, hsa-miR-7852-3p, hsa-miR-6878-3p, hsa-miR-4789-3p, hsa-miR-6747-3p, hsa-miR-4725-5p, hsa-miR-4743-3p, hsa-miR-4649-3p, hsa-miR-4312, and hsa-miR-6733-3p) and their corresponding target mRNAs were depicted. The interaction of hsa_circ_0007507 and these miRNAs was preliminarily verified by dual luciferase assay (**Supplementary Files S5**), indicating that hsa_circ_0007507, which was highly expressed in gastric cancer patients’ tissues, serum and gastric cancer cell lines, might be able to sponge functional mi-RNA and play a role in the occurrence and development of gastric cancer. This provides a new direction for future exploration of the regulatory network involved in hsa_circ_0007507 in GC.

## Discussion

GC is one of the most common tumors. It poses a severe threat to human health due to its insidious onset, ease of metastasis, high degree of malignancy, and poor prognosis. General GC laboratory examinations rely on X-rays and endoscopy (21). These tests can be used to assist the diagnosis of GC, and the final diagnosis of GC must be based on biopsy or cytology, however, these auxiliary methods may miss patients at an early stage. Therefore, it is currently necessary to find relatively non-invasive and convenient GC potential diagnostic tumor markers in clinical practice.

CircRNAs are a subclass of ncRNA that is widely expressed in mammalian cells. Much evidence suggests that circRNAs are mainly produced by precursor messenger RNA (pre-mRNA) through alternative splicing. With the development of high-throughput sequencing technology and bioinformatics, circRNAs have been widely observed in eukaryotic cells. The level of expression of circRNAs has specificity in species, tissue, and time (22). More research reports have shown that circRNAs are involved in the development of malignant tumors, and a group of circRNAs is specifically involved in signal regulation in the process of colorectal cancer (23–25). Moreover, a group of circRNAs was found to be abnormally expressed in hepatocellular carcinoma (HCC) (26–28), enabling the early detection of liver cancer. In this study, hsa_circ_0007507 was selected for follow-up research according to the results of circRNA sequencing of three pairs of GC tissues and circRNA-related databases.

Since circRNAs are connected at the 3’ and 5’ ends through exon or intron circularization to form a complete loop structure, they are not easily degraded by an exonuclease and are more stable than linear RNA (29). Our study revealed that compared with RASA1 mRNA, hsa_circ_0007507 did not degrade significantly after RNA exonuclease treatment, indicating that hsa_circ_0007507 is relatively stable. Besides, miR-335 is down-regulated in GC *via* aberrant promoter hypermethylation, which acts on the target RASA1 gene and participates in the GC process. The increase in the expression of RASA1 mRNA (RASA1 mRNA levels) is closely related to the progress of GC (30). Other reports indicate that RASA1 may be used as a promising biomarker for the early detection of pulmonary sarcomatoid carcinoma (31). Our study confirmed that hsa_circ_0007507 derived from its parent gene RASA1 (up-regulated in GC) has a more stable loop structure and is significantly up-regulated in GC. The trend in the expression between hsa_circ_0007507 and its parent gene RASA1 is the same. This will guide us to continue to study its signal regulation axis in the future.

The ideal tumor marker should have organ specificity. This study confirmed that the expression of hsa_circ_0007507 in the serum of patients with GC has specificity while the expression in colorectal cancer, thyroid cancer, and breast cancer was not statistically significant. ROC analysis verified that the AUC area of hsa_circ_0007507 in distinguishing GC patients from healthy donors was 0.832, which was higher than the AUC of other laboratory markers such as CEA and CA199 in the diagnosis of GC. Moreover, combining hsa_circ_0007507 with other existing tumor markers can produce a maximum AUC value of 0.849. These results indicate that hsa_circ_0007507 can be used as a biomarker with good sensitivity and specificity in GC.

Gastritis, intestinal metaplasia and GC are usually confused in terms of symptoms and need to be distinguished with related tests such as gastroscopy or barium meal. In particular, gastritis and intestinal metaplasia are the main precancerous lesions of GC, developing into GC after multiple stages and accumulation of multi-gene mutations, therefore, the screening of serum markers is an essential method for early diagnosis and early treatment of high-risk groups of gastritis, intestinal metaplasia and GC. In this study, the results showed that, as gastritis developed towards gastric cancer, the expression of hsa_circ_0007507 showed an increasing trend. Thus, hsa_circ_0007507 can be used as an auxiliary screening method for gastritis. Regarding the differences in the expression of other circRNAs between gastritis and otherwise healthy people, Wang et al. found that the expression of hsa_circ_0005654 in 43 gastritis patients was significantly higher than that of healthy donor samples (*p* < 0.001) (32); however, Xu et al. discovered that the expression of hsa_circ_0004771 in 40 patients with gastritis was not significantly different from that of healthy donor samples (*p* = 0.1639) (33). Therefore, not all the expression of circRNAs arises in the serum samples of gastritis patients. In future research, more samples will be collected to verify that hsa_circ_0007507 can be used as a biomarker for gastritis screening.

In the 16 post-operative serum samples we collected, the expression of hsa_circ_0007507 exhibited a significant downward trend. Among the 65 relapsed serum samples and 36 non-relapsed samples we collected, hsa_circ_0007507 exhibited a significant upward trend in recurrence samples. The amount of the recurrence samples we collected was sufficient, exceeding that required to assess statistical significance. In addition, follow-up and survival curves showed that hsa_circ_0007507 could serve as an independent prognostic marker. Therefore, hsa_circ_0007507 can be used as a biomarker for post-operative monitoring, GC recurrence monitoring, and reoperation indications.

Our previous research demonstrated that hsa_circ_0007507 has advantages as a clinical diagnostic marker. We attempt to explore further the relevant mechanism of hsa_circ_0007507 in the development of GC and provide new ideas for GC treatment. This study revealed that the expression of hsa_circ_0007507 was significantly up-regulated in BGC-823, MKN-1, and SGC-7901, and down-regulated in HGC-27, MKN-45, and AGS. The results may be caused by the different characteristics of HGC-27, MKN-45 and AGS cell lines and the different patient sources, leading to the inhibition of hsa_circ_0007507 expression. The nuclear and cytoplasmic separation tests in this study indicated that hsa_circ_0007507 accounted for a high proportion of the cytoplasm, suggesting that it may regulate the process of GC at the post-transcriptional level, which was also confirmed by FISH assay using BGC-823 cell line. Besides, the circRNA-miRNA-mRNA regulation axis in GC was predictable. As illustrated by bioinformatics analysis, hsa_circ_0007507 may interact with hsa-miR-676-5p, hsa-miR-544b, hsa-miR-7852-3p, hsa-miR-6878-3p, hsa-miR-4789-3p, hsa-miR-6747-3p, hsa-miR-4725-5p, hsa-miR-4743-3p, hsa-miR-4649-3p, hsa-miR-4312, and hsa-miR-6733-3p. Among these miRNAs, the transfer of miR-544 mediated by extracellular vesicles of GC inhibits the expression of promyelocytic leukaemia zinc finger (PLZF) protein, increasing the invasion potential (34). Furthermore, Woo et al. found that miR-6733 acted as a GC tumor suppressor and can inhibit the proliferation of GC cells (35). These findings reveal a diverse regulatory network in the GC process, which may involve hsa_circ_0007507.

The expression of hsa_circ_0007507 in GC serum samples was significantly up-regulated, verifying that hsa_circ_0007507 may be a potential diagnostic biomarker for GC. The combined diagnosis of hsa_circ_0007507 and existing immunohistochemical markers can significantly improve diagnostic accuracy; however, the detailed mechanism of action of hsa_circ_0007507 in GC remains to be confirmed, and the circRNA–miRNA–mRNA regulation axis predicted by bioinformatics needs to be further verified in the future, so as to deepen understanding of the role of hsa_circ_0007507 in the progress of GC.

## Data Availability Statement

The datasets presented in this study can be found in online repositories. The names of the repository/repositories and accession number(s) can be found in the article/**Supplementary Material**.

## Ethics Statement

The studies involving human participants were reviewed and approved by the Ethics Committee of Affiliated Hospital of Nantong University (ethical review report number: 2018-L055). The patients/participants provided their written informed consent to participate in this study.

## Author Contributions

WZ completed the conception of the research and the draft of the article. MZ completed the experimental part of the research and analyzed the data. SK provided the source of sequencing and organized the data. XL, and SM were responsible for the collection of experimental samples and the experimental part. XW and FW revised and polished the article. CT and SJ provided financial support for the experiment and grasped the direction and progress of the experiment in the overall situation. All authors contributed to the article and approved the submitted version.

## Funding

This work was supported by the National Natural Science Foundation of China (81871720, 82072363) and PhD Research Startup Foundation of Affiliated Hospital of Nantong University (tdb2011).

## Conflict of Interest

The authors declare that the research was conducted in the absence of any commercial or financial relationships that could be construed as a potential conflict of interest.

## Publisher’s Note

All claims expressed in this article are solely those of the authors and do not necessarily represent those of their affiliated organizations, or those of the publisher, the editors and the reviewers. Any product that may be evaluated in this article, or claim that may be made by its manufacturer, is not guaranteed or endorsed by the publisher.

## Supplementary Material

Supplementary Files S1 Clustered heatmap. Each column represents a tissue sample and each row represents a circRNA. The color reflects the multiple of log_2_ difference ranging from green (low) to black (medium) to red (high). N, normal; C, cancer.

Supplementary Files S2 Volcano plots. Each dot represents a circRNA. Among them, the red points represent significantly up-regulated ones while the green points indicate down-regulation.

Supplementary Files S3 Differential expression of hsa_circ_0007507 in the GC tissues (n=20), and paired adjacent tissues (n=20).

Supplementary Files S4 The FISH assay of hsa_circ_0007507 in BGC-823.

Supplementary Files S5 Luciferase reporter assay was conducted using BGC-823 and MKN-1 cotransfected with hsa_circ_0007507 and predicted miRNAs.

## Abbreviations

GC, gastric cancer; circRNA, circular RNA; AGE, agarose gel electrophoresis; RT-qPCR, real-time fluorescent quantitative polymerase chain reaction; ROC, receiver operating characteristic curve; AUC, area under the ROC; CI, confidence interval; ncRNA, non-coding RNA; 1ncRNA, long-chain non-coding RNA; HCC, hepatocellular carcinoma; ecRNA, exon circRNA; ciRNA, intron circRNA; EIciRNA, exon-intron circular RNA; RNase R, ribonuclease R; gDNA, genomic DNA; GAPDH, glyceraldehyde 3-phosphate dehydrogenase; OS, overall survival.
