# Supplementary figures and images for: Circular RNA hsa_circ_0007507 May Serve as a Biomarker for the Diagnosis and Prognosis of Gastric Cancer
Source: Front Oncol. 2021 Sep 14;11:699625. doi: 10.3389/fonc.2021.699625 (PMC8477006; doi:10.3389/fonc.2021.699625)

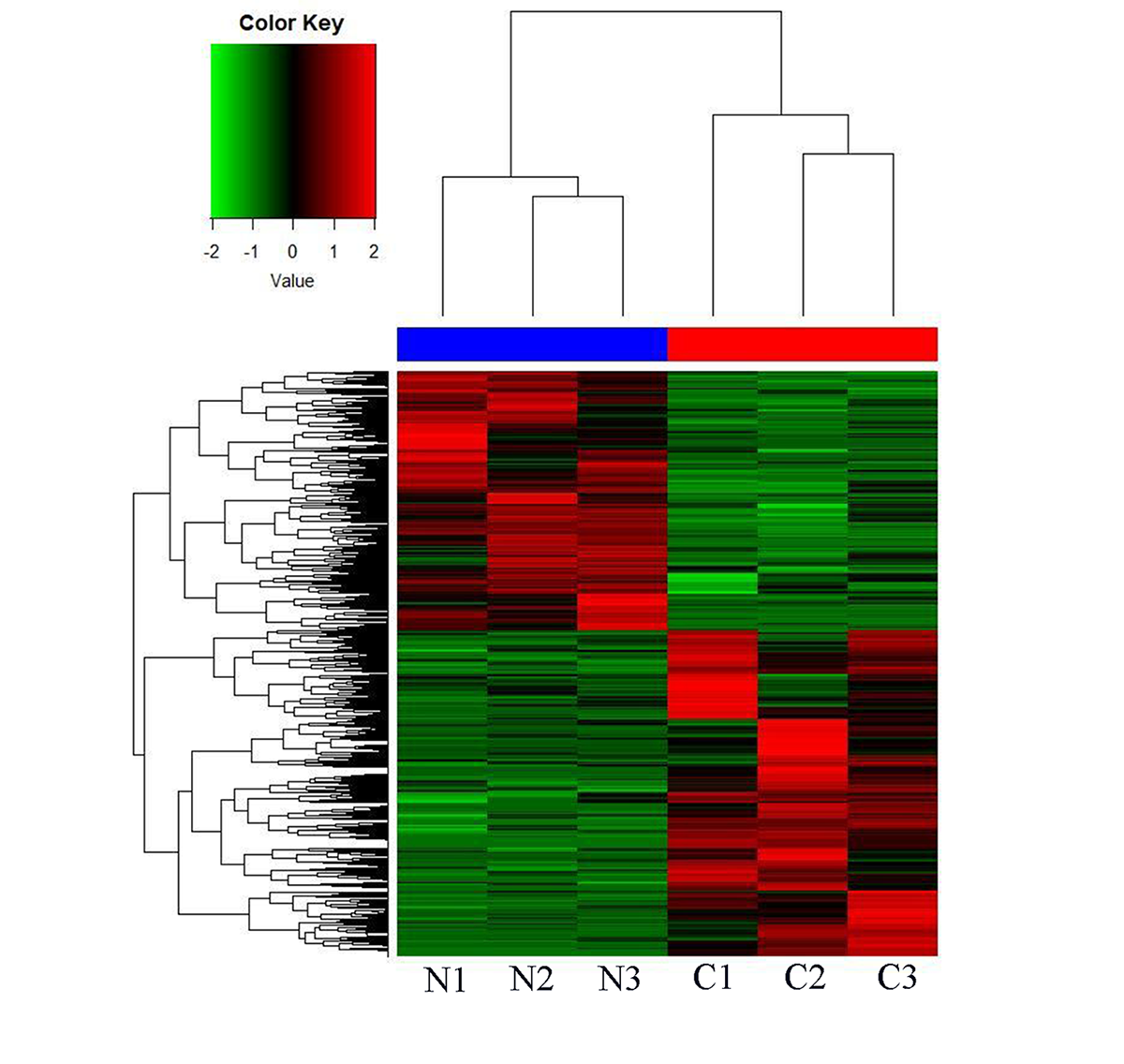

Supplement: Supplementary Files S1 — Clustered heatmap. Each column represents a tissue sample and each row represents a circRNA. The color reflects the multiple of log2 difference ranging from green (low) to black (medium) to red (high). N, normal; C, cancer. [file Image_1.tif]

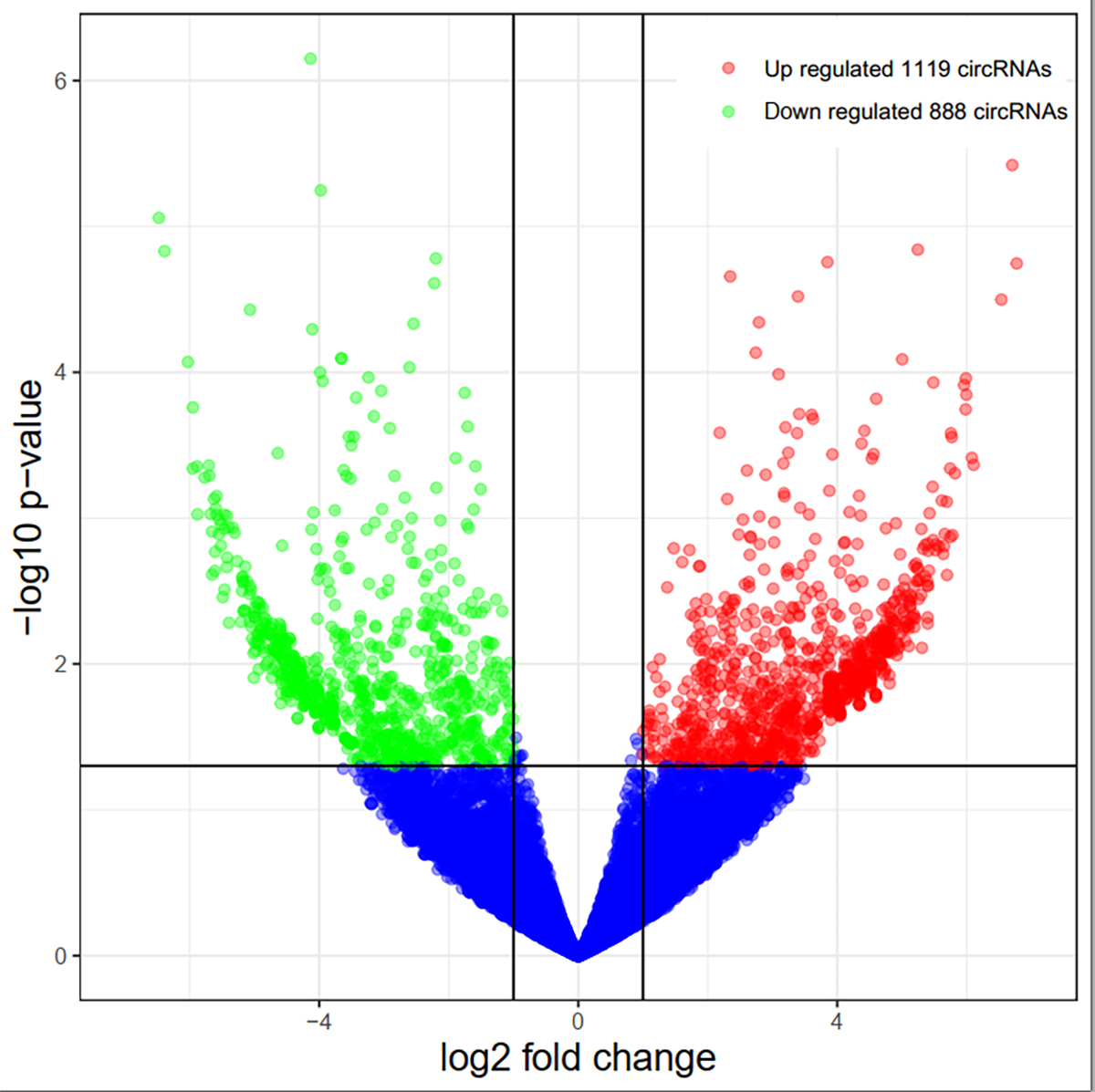

Supplement: Supplementary Files S2 — Volcano plots. Each dot represents a circRNA. Among them, the red points represent significantly up-regulated ones while the green points indicate down-regulation. [file Image_2.tif]

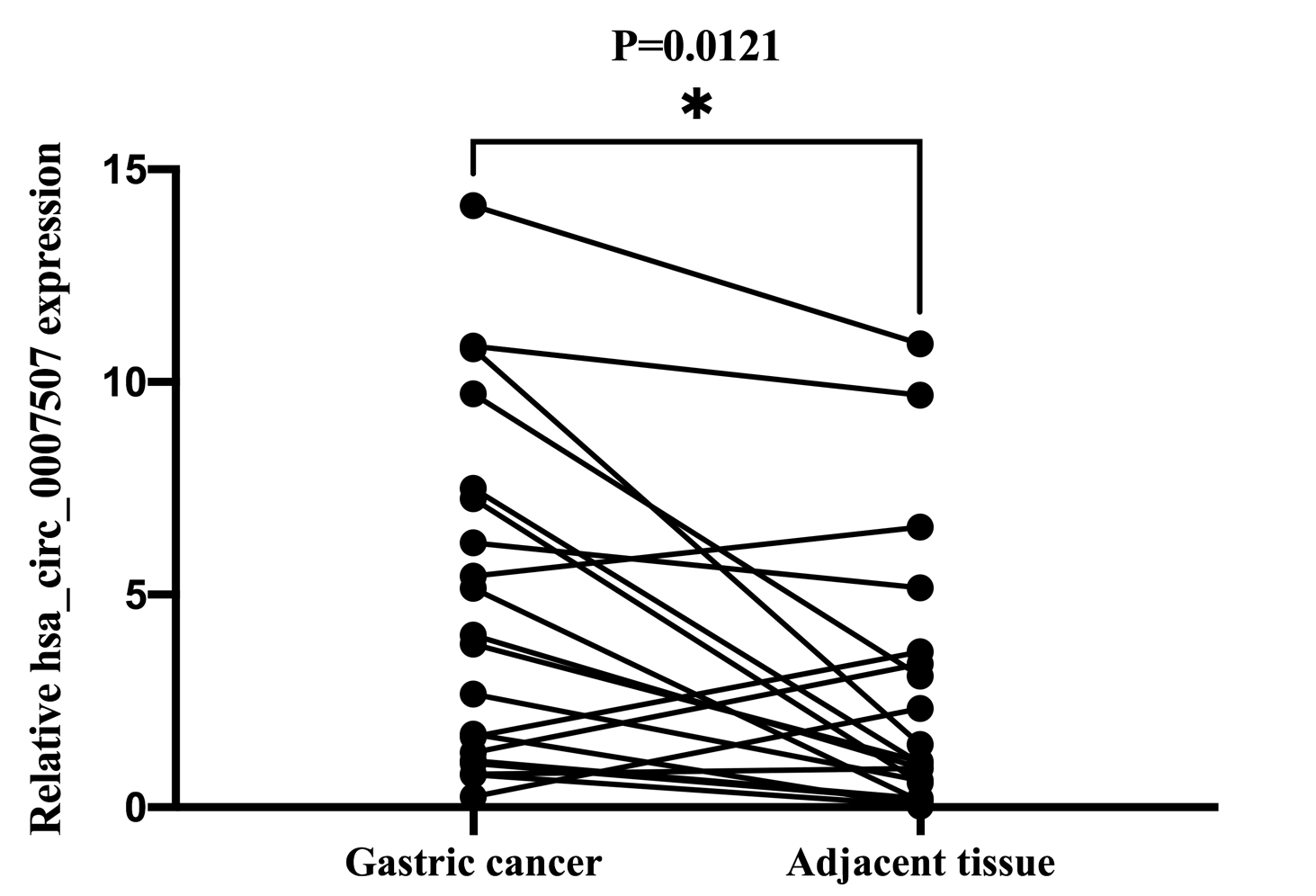

Supplement: Supplementary Files S3 — Differential expression of hsa_circ_0007507 in the GC tissues (n=20), and paired adjacent tissues (n=20). [file Image_3.tiff]

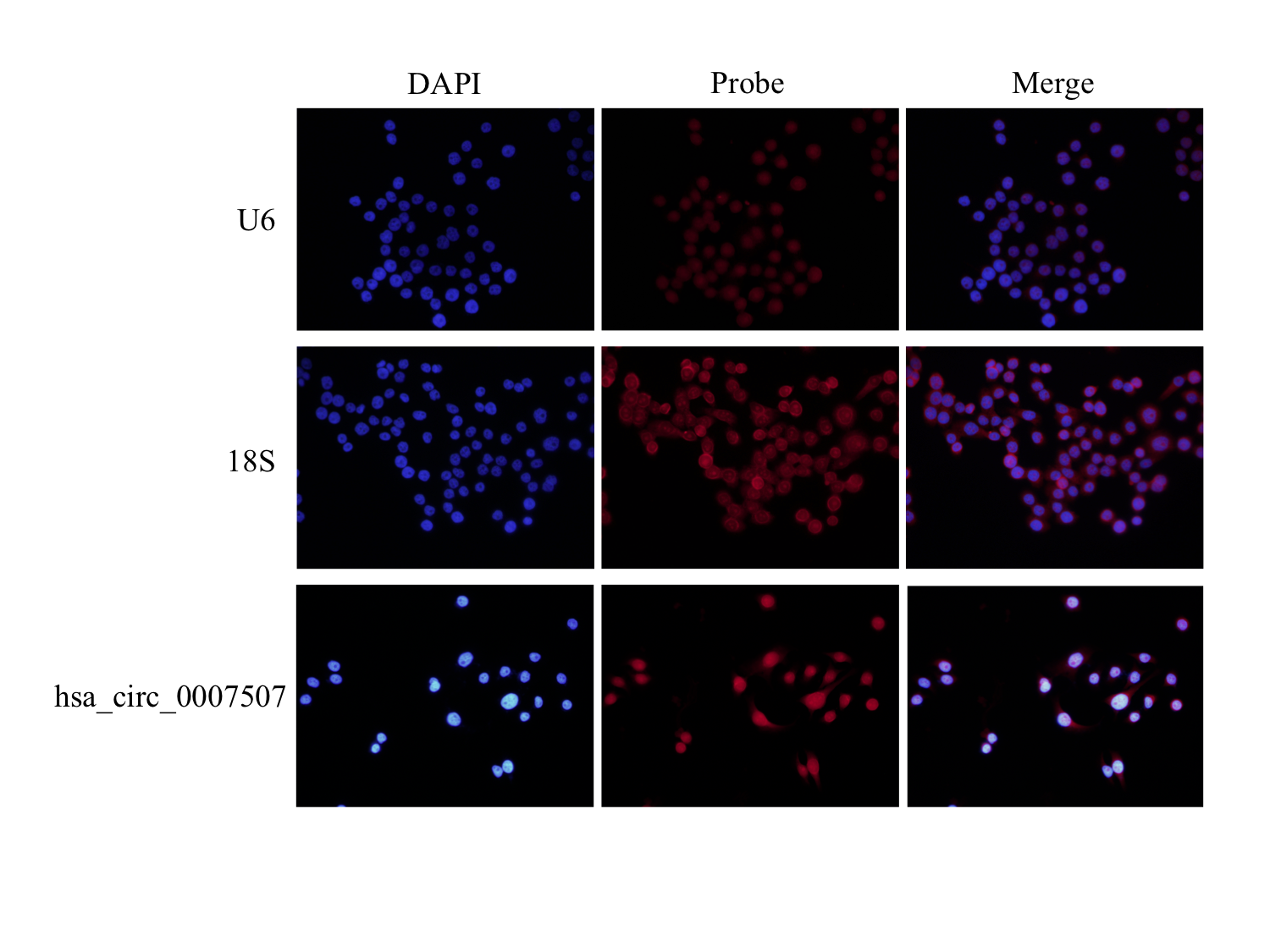

Supplement: Supplementary Files S4 — The FISH assay of hsa_circ_0007507 in BGC-823. [file Image_4.tiff]

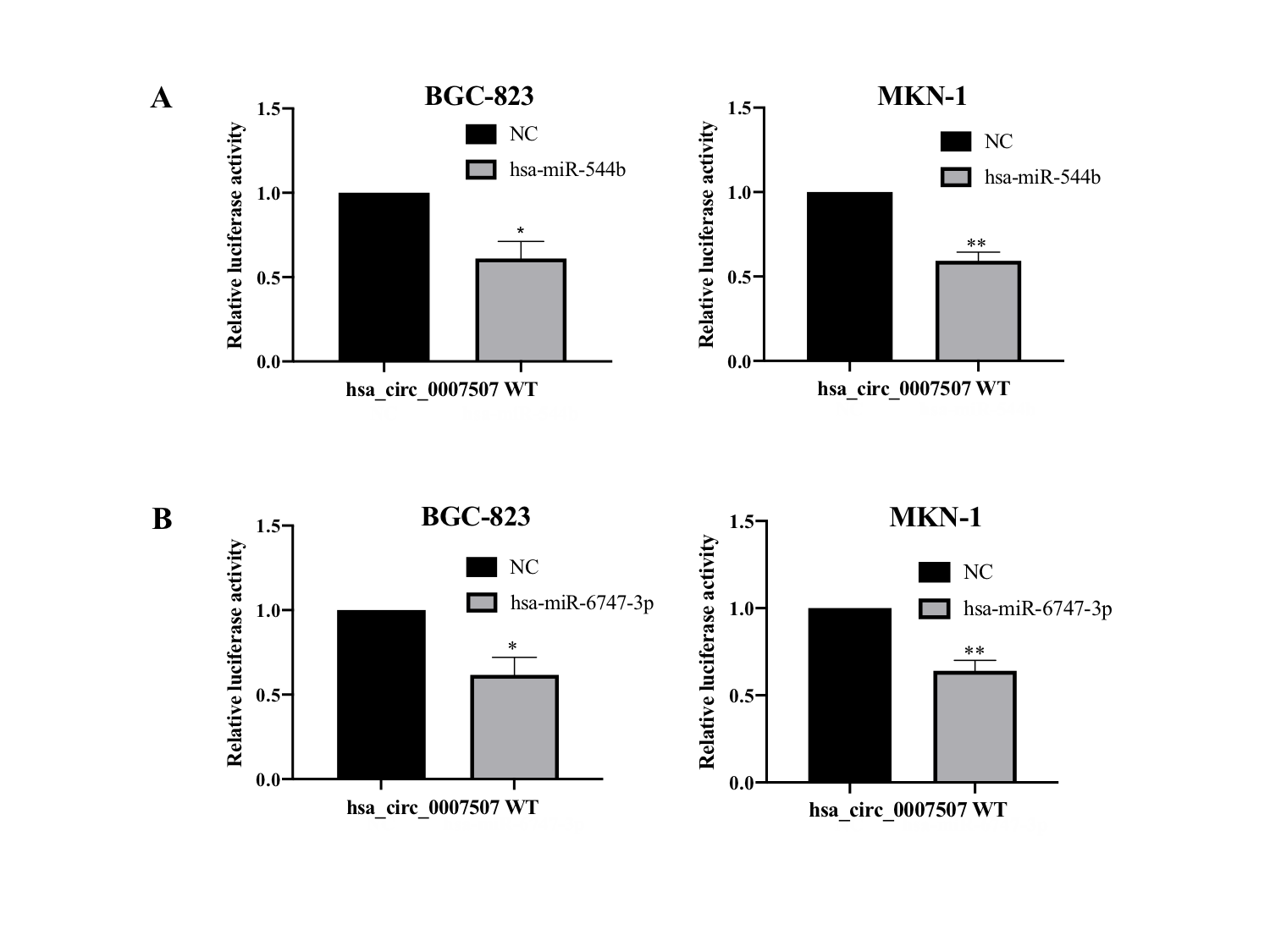

Supplement: Supplementary Files S5 — Luciferase reporter assay was conducted using BGC-823 and MKN-1 cotransfected with hsa_circ_0007507 and predicted miRNAs. [file Image_5.tiff]
